# Effects of transgenerational photoperiod experience on the reproduction and development of *Anastatus orientalis*, an egg parasitoid of the spotted lanternfly

**DOI:** 10.3389/finsc.2023.1153723

**Published:** 2023-05-17

**Authors:** Ke-xin Bao, Xiao-yi Wang, Liang-ming Cao, Bei Xin, Hannah J. Broadley, Juli R. Gould

**Affiliations:** ^1^ Key Laboratory of Forest Protection of National Forestry and Grassland Administration, Ecology and Nature Conservation Institute, Chinese Academy of Forestry, Beijing, China; ^2^ College of Forestry and Landscape Architecture, Xinjiang Agricultural University, Urumqi, China; ^3^ Forest Pest Methods Laboratory, United States Department of Agriculture, Animal and Plant Health Inspection Service, Plant Protection and Quarantine, Science and Technology, Buzzards Bay, MA, United States

**Keywords:** *Anastatus orientalis*, diapause, *Lycorma delicatula*, maternal effect, parasitoid, photoperiod, transgenerational effect

## Abstract

Transgenerational experience can affect a range of natural enemies’ life-history traits and can be involved in the control of developmental plasticity. As a major egg parasitoid of the spotted lanternfly, *Lycorma delicatula* (Hemiptera: Fulgoridae), the wasp *Anastatus orientalis* (Hymenoptera: Eupelmidae) is effective at suppressing its host populations. The reproductive and developmental traits of *A. orientalis* is known to depend on photoperiod conditions, but transgenerational photoperiodic effects have yet to be evaluated. To evaluate the transgenerational photoperiodic effects on *A. orientalis*, we assessed wasp adult longevity, female fecundity, sex ratio, and diapause rate over three consecutive generations under different experimental photoperiods (L16:D8, L12:D12, and L8:D16), using *Antheraea pernyi* (Lepidoptera: Saturniidae) eggs as hosts. The results suggest that transgenerational experience significantly impacts several biological parameters of progeny. All parasitoids entered a diapause under the long photoperiod condition (i.e., L16:D8), after which the number of female parasitoids and fecundity of the 2nd and 3rd generations increased significantly as compared to the 1st generation. With the long photoperiod conditions, the female ratio rose from 68.1% (1st generation) to 86.0% (3rd generation) and the progeny per females increased from 35.8 to 75.7. However, adult longevity of females and males were shortened significantly. With the intermediate photoperiod (L12:D12) conditions, fecundity and sex ratio of the 2nd and 3rd generations increased significantly as compared to the 1st generation. With the short photoperiod (L8:D16) conditions, there were no significant differences in fecundity among three generations, but sex ratio of the 2nd and 3rd generations increased significantly as compared to the 1st generation. These results on transgenerational photoperiodic effects can be applied to improve laboratory rearing efficiency of parasitoids and to better understand population dynamics in the field across a latitudinal gradient.

## 1 Introduction

Extensive phenotypic plasticity can allow the populations of natural enemies to better adapt to changes in local environmental conditions and thereby gain greater efficiency in attacking their target pests. Phenotypic plasticity includes transgenerational effects, which are known to affect natural enemies’ reproductive and developmental traits, such as diapause, survival rate, development time, and oviposition (1–4). Although not all plastic responses of natural enemies are optimal, phenotypic plasticity is particularly advantageous when the environmental conditions the progeny will face can be better predicted by the parents than by the progeny themselves. For example, cues experienced by parents can provide progeny with additional environmental information beyond their own and the parents can induce or inhibit diapause in their progeny and affect other traits depending on the environmental signals they perceive (5–8). Transgenerational effects can be broader than simply maternal effects, and mounting evidence suggests that a grandmaternal effect can also alter the fecundity and development of natural enemies (9–12). Yet controlled evaluations of grandmaternal effects are limited and our understanding of how grandmaternal effects enable the progeny to better adapt to changes in their environmental conditions is still not well explained.

The spotted lanternfly, *Lycorma delicatula* White (Hemiptera: Fulgoridae), is a recent invasive pest in South Korea, Japan, and North America (13–16), where it threatens commercially grown grapevines and tree fruits, in addition to plant nurseries and timber industries (17, 18). Despite intensive efforts by federal, state, and local stakeholders to stop the spread of spotted lanternfly in the USA, the populations continue to spread. Since initial detection of spotted lanternfly in Berks County, Pennsylvania, USA, in 2014, spotted lanternfly infestations have been detected in 130 counties (87 under quarantine) within Connecticut, Delaware, Indiana, Maryland, New Jersey, New York, Ohio, Virginia, and West Virginia (19). Additional control methods are needed to help in the management of spotted lanternfly populations in the USA, and we are evaluating important parasitoids of spotted lanternfly in its native range as candidate biological control agents.

The insect of the current study, the wasp *Anastatus orientalis* (Hymenoptera: Eupelmidae), is the most widespread native egg parasitoid of spotted lanternfly, and has been used as a biological control agent in South Korea and is being considered a candidate for biological control in the USA (20–23). Large numbers of wasps are needed for testing candidate biological control agents or implementing a biocontrol programme, but artificial rearing spotted lanternfly is difficult, and the methods are not well developed (24). Thus, for laboratory research in South Korea on *A. orientalis*, eggs of a substitute host *Antheraea pernyi* (Lepidoptera: Saturniidae) were used for rearing the parasitoid (25). For this study, owing to the challenges of rearing or acquiring non-parasitized spotted lanternfly egg masses, we are also using *A. pernyi* as the host for rearing. However, when using substitute hosts to rear a parasitoid, changes in the biological characteristics of the insects under different rearing conditions should be considered because non-target hosts could influence the parasitoids’ response to the target host. They could influence offspring sex or vitality and poor-quality parasitoids may yield low efficiency in reared colonies or biological control programmes (26–28). Suitable environmental conditions for both the development and fecundity of natural enemies are imperative for their mass rearing in laboratory settings. Photoperiod and temperature are widely considered the most influential external factors for the development and fertility of natural enemies (29, 30).

Evaluations of the life cycle of *A. orientalis* in the field in China indicate that adults had two emergence periods per year. Some individuals emerged in May and others in September, with a summer diapause in between (21). Hou (31) found that, in a laboratory setting, *A. orientalis* could complete seven or eight generations over a span of 7 months (reared from April to December) when reared at 25°C with a L12:D12 photoperiod, and the emergence rate could reach up to 78%. In contracts, Broadley et al. (23) observed a low emergence rate of *A. orientalis* exposed to 16L:8D hour long-day conditions at 25°C, with most parasitoids entering a diapause. This suggests that summer diapause in *A. orientalis* is induced by a long photoperiod. In another study, Seo et al. (22) reported the longevity, oviposition, and sex ratio of *A. orientalis* under a 16L:8D photoperiod at four different temperatures (15°C, 20°C, 25°C, and 30°C). This led those authors to speculate that photoperiod influences the reproductive and developmental traits of *A. orientalis*. Thus, understanding the relationship between photoperiod and natural enemies’ biological characteristics is crucial for optimizing rearing methods (32, 33).

A transgenerational effect has been observed in other *Anastatus* species. In addition to environmental variables, the maternal oviposition experience and age of *Anastatus disparis* (Hymenoptera: Eupelmidae), an egg parasitoid of *Lymantria dispar* (Lepidoptera: Lymantriidae), significantly influences sex allocation in their progeny (34, 35). To our knowledge, however, no study has yet reported on whether or not transgenerational experience affects the biological characteristics of *A. orientalis.* Moreover, the maternal photoperiodic response has not been sufficiently investigated. Hence, in the present study, we investigated the effects of transgenerational photoperiod experience on the fertility and development of *A. orientalis* by using *A. pernyi* eggs as a host. The empirical data on the effects of transgenerational photoperiod experience on insect fertility and development acquired by our study could also be used for optimizing rearing methods and storage of natural enemy parasitoids, and for better understanding *A. orientalis* population dynamics in the field across a geographic and climatic range.

## 2 Materials and methods

### 2.1 Insect rearing

A population of *A. orientalis* was collected from overwintering spotted lanternfly egg masses in Haidian (40˚00′34′′N, 116˚23′32′′E), Beijing, China. The spotted lanternfly egg masses were kept under laboratory conditions at 25 ± 1°C and 60% ± 5% relative humidity (RH), with a L12:D12 photoperiod until parasitoid emergence. The parasitoid species were confirmed using scanning electron microscopy (SEM) micrographs by examining the morphology of adult specimens (21). The *A. orientalis* were reared for five generations in 25°C with 12 h of light using *A. pernyi* eggs as their host (25), and then they were reared another three generations on *A. pernyi* in 25°C with 14 h of light (22). These conditions were identified as effective rearing conditions for *A. orientalis*. Within the first 48 h after emergence, five females and one male of *A. orientalis* (i.e., ‘Generation 0’, G0) were placed in ventilated insect rearing cages (20 cm × 20 cm × 20 cm) for 36 h for mating, with access to honey and water.

For each experiment, a kraft paper card with 100 A*. pernyi* eggs glued to it (with a polyvinyl acetate suspension) were prepared. We selected one female parasitoid (G0), which was released into a transparent plastic container (a 482-ml plastic deli cup) with one *A. pernyi* egg card each to obtain the first progeny generation (‘Generation 1’, G1) of *A. orientalis*. Then, the containers were randomly separated into three treatment groups that were placed in three photoperiodic regimes: L:D = 8:16, 12:12, or 16:8 at 60%–75% RH, at 25°C. After a 48-h exposure to the egg card, female adult parasitoids were removed, while the parasitized eggs were maintained at different photoperiods conditions until emergence from the eggs.

**Figure 1:**
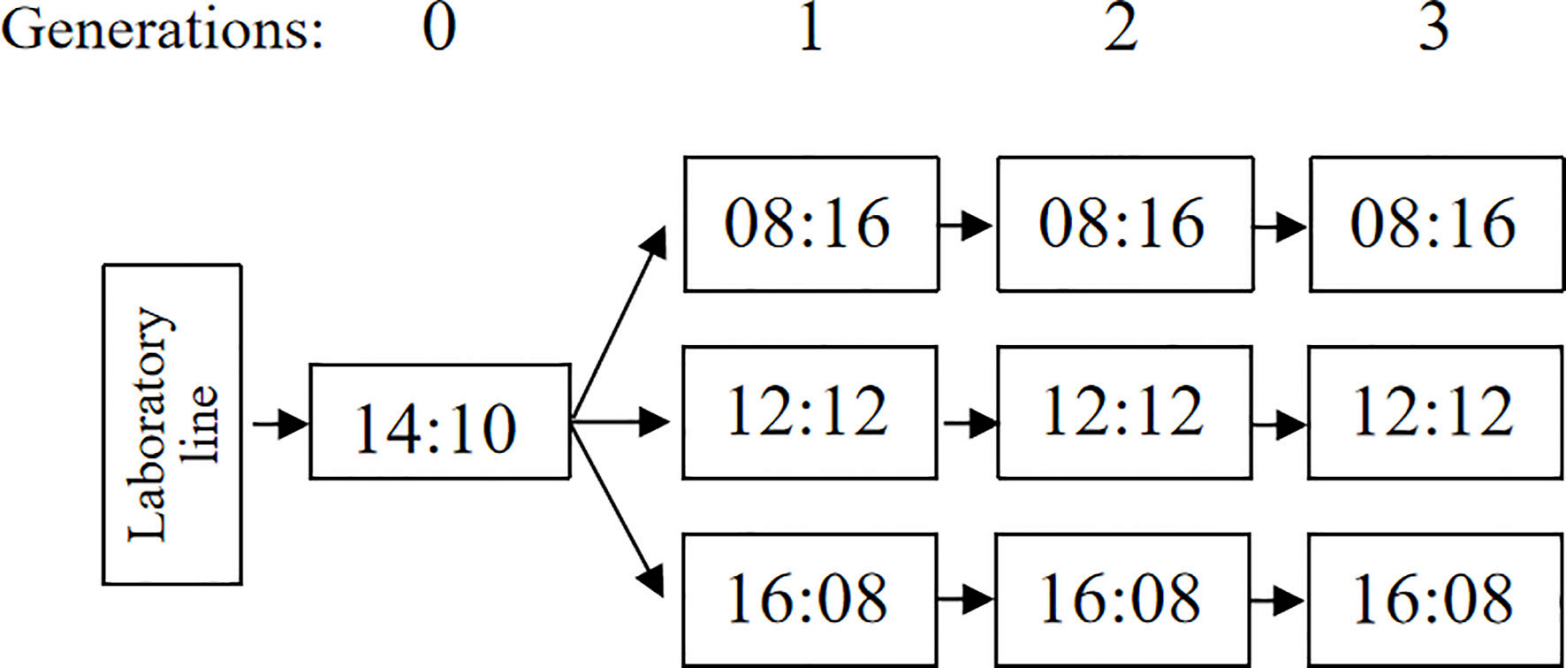
The general design of the experiment. Generations are indicated at the top of the figure, photoperiods are shown in the figure as number of hours light:dark, and the arrows indicate the succession of generations.

To obtain the second and third generations (‘Generation 2’, G2; ‘Generation 3’, G3) of *A. orientalis* in three photoperiodic regimes, five females and one male less than 48 h old were placed in a ventilated insect rearing cage (20 cm × 20 cm × 20 cm container, described above) under each rearing condition, these were following generations all reared in one of the three photoperiod treatments the same as their parent generations. In the meantime, honey and water were provided in each condition for 36 h for mating success. Then, one mated female *A. orientalis* was selected randomly and placed in a transparent plastic container (a 482-ml container, described above) with one new paper card with 100 fresh eggs of *A. pernyi*. After a 48-h exposure to fresh eggs of *A. pernyi*, the female *A. orientalis* adult was removed, while the parasitized eggs were kept in the same rearing condition until emergence from eggs (**Figure 1**).

### 2.2 Observation of fecundity, sex ratio, adult longevity, and diapause pattern of the parasitoid wasp

To determine the fecundity, sex ratio, and diapause of the parasitoid wasps of different generations under three photoperiods, 40 days after the mass emergence of the non-diapausing fraction of the progeny generation, all parasitized host eggs were dissected and examined, respectively. If none of the parasitoid progeny emerged under a certain treatment, they were considered to be in a diapause state. Therefore, to obtain parasitoid progeny, the host eggs were not dissected until wasp emergence after approximately 3 months. The diapausing larvae (each living larva that was assumed to be in diapause) and non-diapausing individuals (mostly emerged adults, few dead adults inside the host, and sporadic pupae) were identified and counted, and the sex of the progeny that successfully emerged from each egg mass was recorded. Because typically only one *A. orientalis* is produced out of each *A. pernyi* egg, the number of emerged adults was estimated as the number of parasitized eggs with emergence holes. The few (less than 1%–2%) individuals that died during the larval or prepupal stages were excluded. Next, the percentage of diapausing individuals was calculated for each generation in each photoperiod condition. To determine the percentage diapausing, a random sample of 100 host eggs that were parasitized during a 48-h window by one female were dissected. Overall, the experiment included 10 replicates for each of the three photoperiod treatments conducted with each of the three generations of *A. orientalis* (for a total of 90 cards and 9,000 parasitized host eggs). To estimate the average longevity of *A. orientalis* adults of different generations under three photoperiods, newly emerged (< 24 h old) *A. orientalis* of each treatment were collected. Each adult was kept individually in a rearing container (a 482 ml-container, described above) in each photoperiod condition, with 10% honey. The wasps were checked daily, and the date of death was recorded until all individuals died.

### 2.3 Statistical analyses

Excel 2007 and SPSS 19.0 software programs were used to statistically analyze the data. Two-way ANOVA followed by least significant difference tests was used to compare longevity of adult and female fecundity among photoperiods, generation, and their interaction (Tukey’s *post-hoc* test, *p* < 0.05). To further evaluate the effects of photoperiod and generation on daily survival of *A. orientalis*, effects of different treatments on the adult longevity of *A. orientalis* were represented by Kaplan–Meier survival curves. Survival time was measured in days from the date of experiment beginning to the date of wasp death. Each adult was used as one replicate. A mixed-effects Cox regression model was fitted to analyze the death risk of *A. orientalis* reared from different treatments, including generations, photoperiods, and longevity as covariates, using the package “coxme” in SPSS software. We present a hazard ratio with corresponding 95% CI. The differences in the sex ratio and diapause between different photoperiods or generations were compared using chi-squared tests. We also examined potential trade-offs between life history traits by performing linear regression analyses between diapause trait and other traits.

## 3 Results

### 3.1 Female fecundity

**Figure 2:**
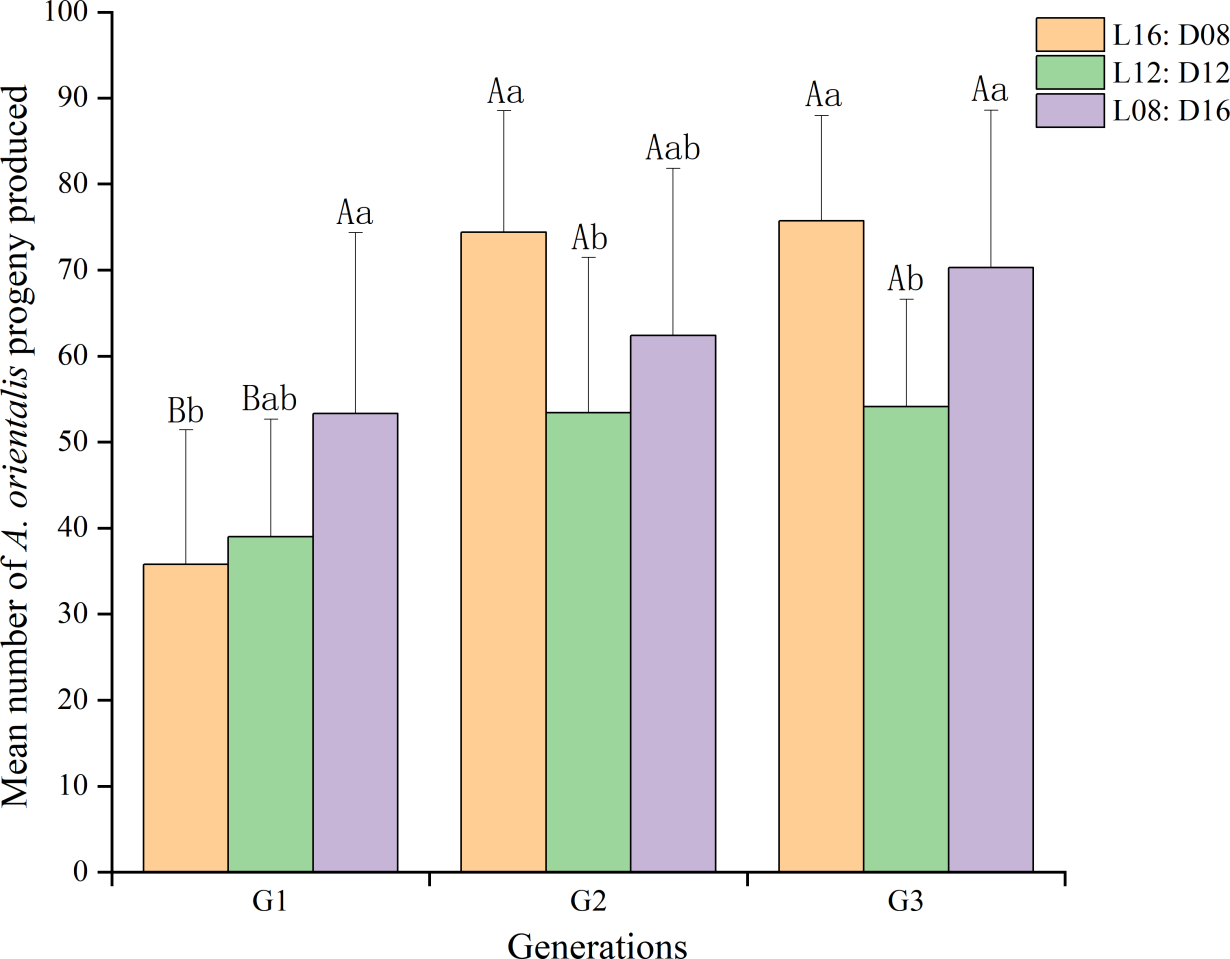
Effect of generation on the female fecundity of *Anastatus orientalis* under three photoperiods. Different upper-case letters within the same photoperiod indicate significant differences among the three generations, whereas different lower-case letters within the same generation represent significant differences among the three photoperiods (one-way ANOVA, least significant difference multiple comparison, *p* < 0.05). The bars show means and standard deviation.

We found that generation significantly affected (df = 2, 81; *F* = 18.914; *p* < 0.01) female fecundity, which increased in later generations under the same photoperiod (**Figure 2**). Under the photoperiod L16:D8, the mean number of *A. orientali*s progeny produced was larger in the second and third generations than in the first generation (74.40, 75.70, and 35.80, respectively), and the differences were significant (df = 2, 27; *F* = 26.015; *p* < 0.01). Under the photoperiods L12:D12 (df = 2, 27; *F* = 3.253; *p* = 0.054) and L8:D16 (df = 2, 27; *F* = 1.883; *p* = 0.172), the progeny produced were similar among the first, second, and third generations.

Fecundity outcomes of *A. orientali*s females of the same generation under each of the three photoperiods are plotted in **Figure 2**. The photoperiod also affected (df = 2, 81; *F* = 6.442; *p* < 0.05) female fecundity, and the interaction between generational and photoperiod was significant (df = 4, 89; *F* = 2.818; *p* < 0.01). For the first generation, their mean number of progenies produced was 53.30 under the L8:D16 photoperiod, this was larger than that produced under the L12:D12 or L16:D8 photoperiods (df = 2, 27; *F* = 2.984; *p* = 0.068). For the second generation, there was a significant difference (df = 2, 27; *F* = 3.685; *p* < 0.05) in female fecundity between L16:D8 (74.40%), L12:D12 (53.4%), and L8:D16 (62.4%) photoperiods. In addition, for the third generation, female fecundity was significantly greater (df = 2, 27; *F* = 5.932, *p* < 0.01) under the L16:D8 (75.70) and L8:D16 (70.30) photoperiods than under the L12:D12 (54.10) photoperiod.

### 3.2 Sex ratio

**Figure 3:**
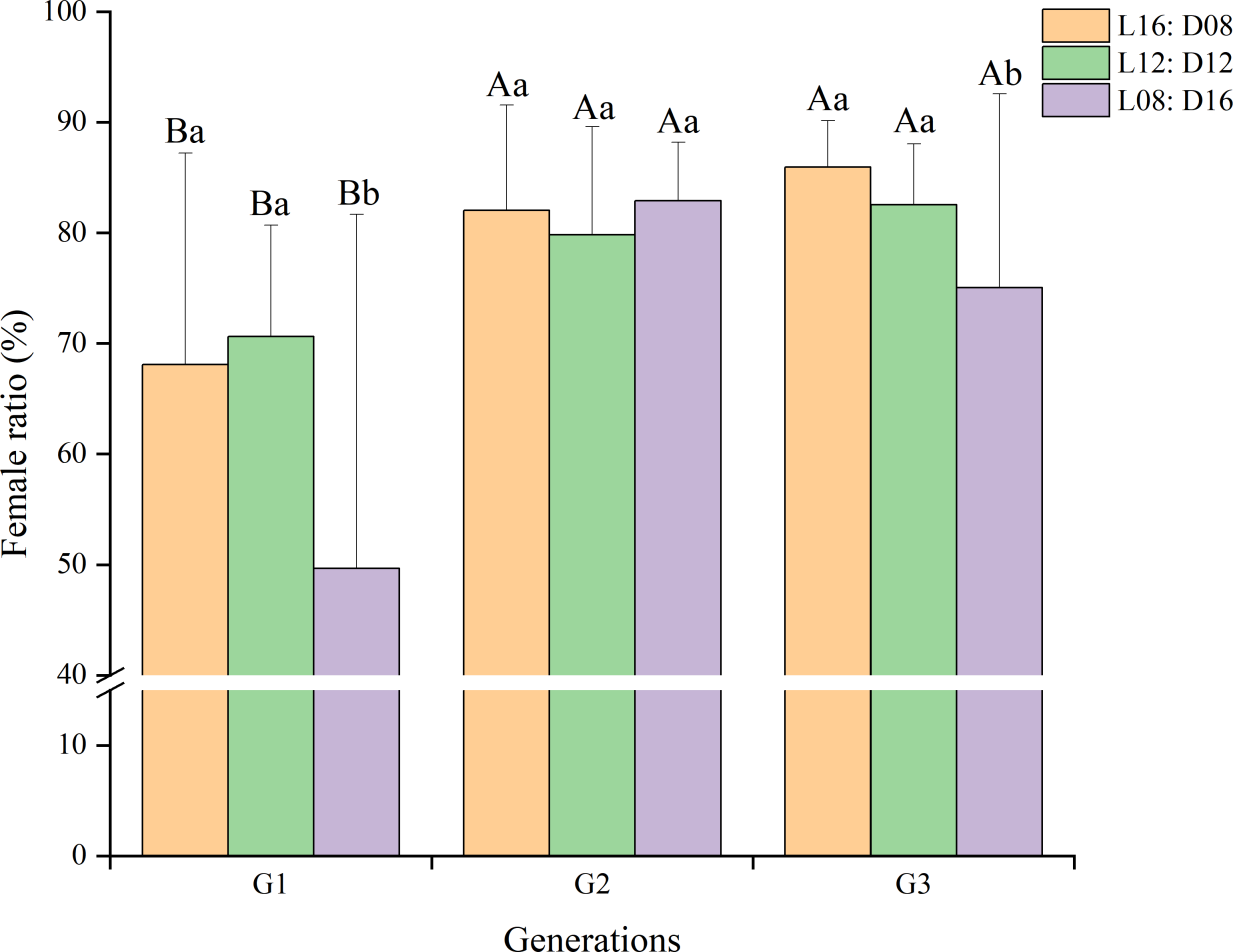
Effect of generation on sex ratio of *Anastatus orientalis* under three photoperiods. Different upper-case letters within the same photoperiod indicate significant differences among the three generations, whereas different lower-case letters within the same generation represent significant differences among the three photoperiods (chi-squared test, *p <* 0.05). The bars show means and standard deviation.

Photoperiod and generation both had a significant effect on sex ratio (photoperiod: *χ* ^2^ = 87.752, df = 2, *p* < 0.001; generation: *χ* ^2^ = 237.355, df = 2, *p* < 0.001). The lowest female ratio was recorded in the first generation, averaging 62.80% across different photoperiods (**Figure 3**). The highest female ratio was recorded in the second generation, averaging 81.59% across different photoperiods. Considering just the third generation, the female ratio of *A. orientalis* was highest (85.96%) under a photoperiod of L16:D8.

### 3.3 Adult longevity

**Figure 4:**
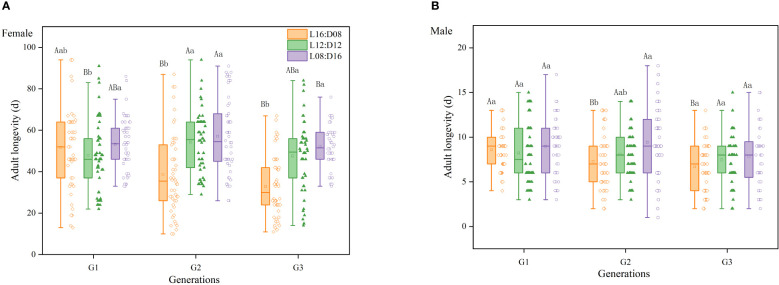
Female **(A)** and male **(B)** longevity of *Anastatus orientalis* wasps from different generations under three photoperiods. Different upper-case letters within the same photoperiod indicate significant differences among the three generations, whereas different lower-case letters within the same generation represent significant differences among the three photoperiods (one-way ANOVA, least significant difference multiple comparison, *p* < 0.05).

The interaction between generation and photoperiod had a significant effect on female longevity (df = 4, 418; *F* = 5.846; *p* < 0.01; **Figure 4A**). However, there was no statistically significant interaction between generation and photoperiod on male longevity (df = 4, 381; *F* = 1.111; *p* = 0.351; **Figure 4B**), and the effects of generation and photoperiod on male longevity were additive rather than synergistic. Photoperiod had a significant effect on female (df = 2, 424; *F* = 29.234; *p* < 0.01) and male (df = 2, 387; *F* = 4.897; *p* < 0.01) longevity. Male and female longevity was longest under the L8:D16 photoperiod (average: 8.69 days for males and 54.50 days for female) and shortest under the L16:D8 photoperiod (average: 7.46 days for males and 39.66 days for females), indicating that a greater photoperiod reduced the longevity of *A. orientalis*. Generation significantly affected female (df = 2, 424; *F* = 5.382; *p* < 0.01) and male (df = 2, 387; *F* = 6.382; *p* < 0.01) longevity, which decreased by increasing generation in the second and third generations of progeny.

**Figure 5:**
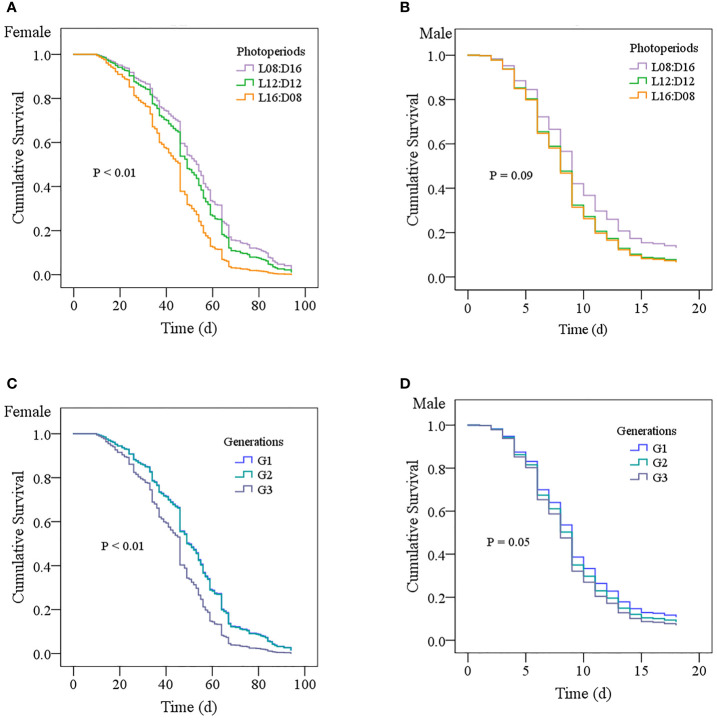
Kaplan–Meier survival curves show the female **(A)** and male **(B)** longevity of *Anastatus orientalis* wasp probabilities over time for the long photoperiod condition L16:D8 (N_female_ = 145, N_male_ = 133), middle photoperiod condition L12:D12 (N_female_ = 143, N_male_ = 133), and short photoperiod condition (N_female_ = 139, N_male_ = 124) lines, as well as by generation **(C, D)**.

The longevity of surviving female wasps under different photoperiod conditions was significantly different (log-rank *p* < 0.01; **Figure 5A**), but male longevity was not significantly different (log-rank *p* = 0.09; **Figure 5B**). The Kaplan–Meier survival curve of female wasps with the photoperiod L16:D08 was consistently below the survival curve of those rearing under the photoperiods L12:D12 and L08:D16. Survival curves of female longevity also showed a significant difference between different generations (log-rank *p* < 0.001; **Figure 5C**), whereas male longevity was not different between different generations (log-rank *p* = 0.05; **Figure 5D**). The Kaplan–Meier survival curve of G3 females was consistently below the survival curve of G1 and G2 females. The hazard ratio of death of females and males for the different photoperiods were 1.20 (95% CI 0.95 to 1.51) and 1.30 (95% CI 1.02 to 1.66) in the mixed-effects Cox regression model, respectively. The hazard ratio of death of females and males for the different generations were 1.01 (95% CI 0.80 to 1.28) and 0.84 (95% CI 0.66 to 1.07), respectively.

### 3.4 Diapause in progeny

**Figure 6:**
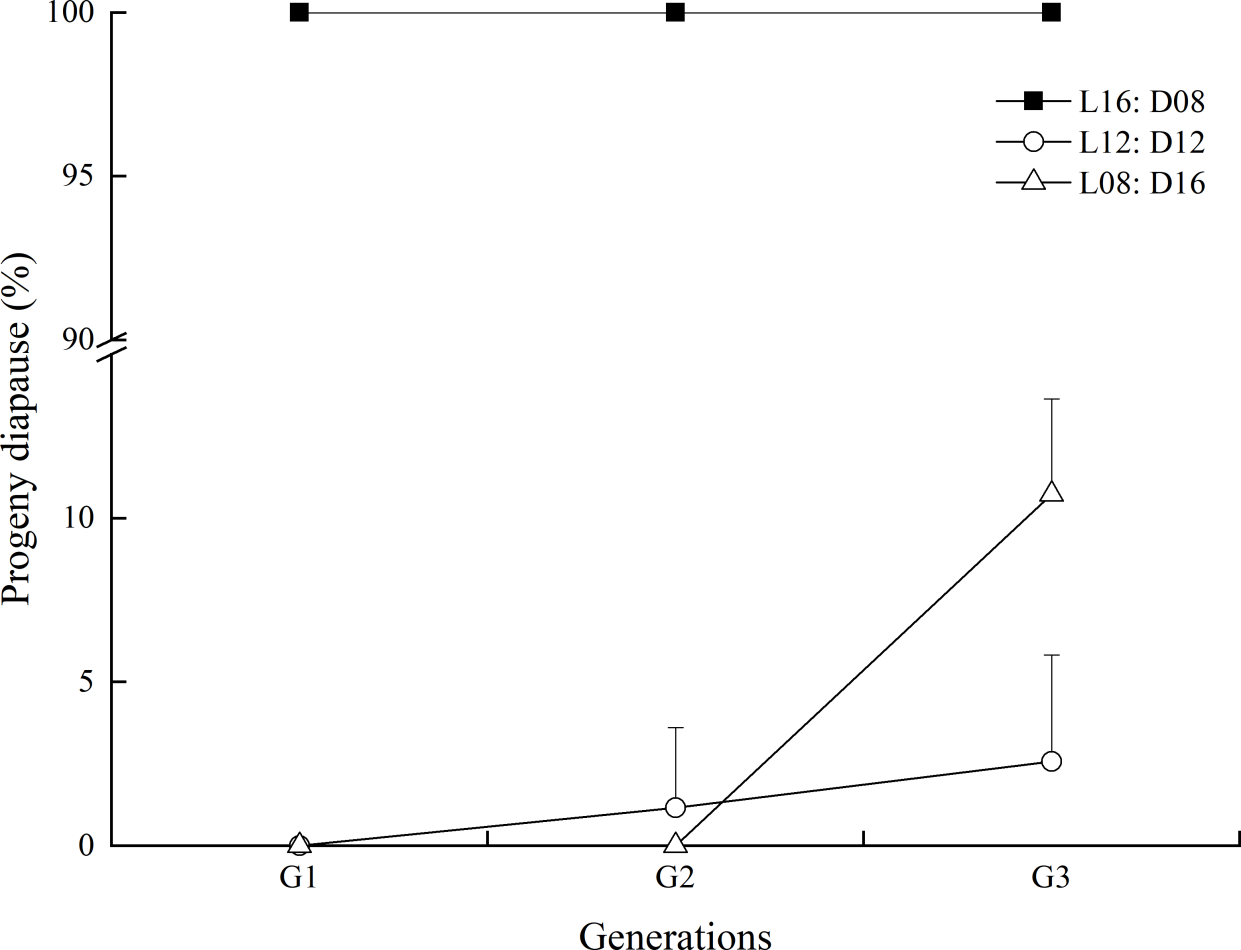
Influence of photoperiod conditions during the development of the transgenerational generations on the diapause of *Anastatus orientalis*. The bars show means and standard deviation.

Photoperiod was the most important factor determining the proportion of diapausing progeny (χ^2 =^ 5820.070, df = 2, *p* < 0.001; **Figure 6**), and the different generations were also significantly influenced (χ^2 =^ 93.602, df = 2, *p* < 0.001). The progeny of *A. orientalis* females that developed under the long-day length entered diapause significantly more often than females that developed under the medium- and short-day lengths. All parasitoids entered a diapause under the long photoperiod condition (L:D = 16:8), irrespective of which generation they constituted. Under the L12:D12 photoperiod, diapause incidence was significantly increased by an increasing generation (χ^2 =^ 28.660, df = 2, *p* < 0.001). Under the short photoperiod (L8:D16), no parasitoid from both the first and second generations entered diapause, but diapause incidence strongly increased for the third generation (χ^2 =^ 143.668, df = 2, *p* < 0.001).

**Figure 7:**
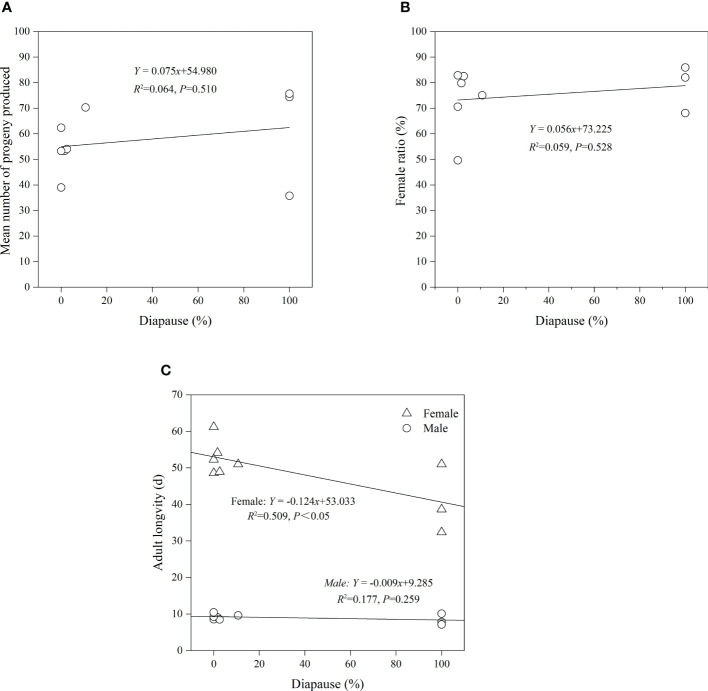
Regression relationship between fecundity **(A)**, female ratio **(B)**, adult longevity **(C)**, and diapause of *Anastatus orientalis*.

There was no significant relationship between diapause of *A. orientalis* and fecundity (*p* = 0.510, *R* ^2^ = 0.064; **Figure 7A**), sex ratio (*p* = 0.528, *R* ^2^ = 0.059; **Figure 7B**), or male longevity (*p* = 0.259, *R* ^2^ = 0.177; **Figure 7C**). There was a positive correlation between diapause and female longevity (linear regression using female longevity and diapause as a dependent and explanatory variable, respectively: *p* < 0.05, *R* ^2^ = 0.509; **Figure 7C**).

## 4 Discussion

Transgenerational photoperiod effects have been shown to influence reproductive and developmental traits of wasp progeny, thus achieving a cumulative photoperiodic effect (34). However, as far as we know, a transgenerational photoperiod influence on the fertility and development of *Anastatus* species progeny has not been clearly demonstrated. The present results show that photoperiod and generation significantly affect female fecundity, female ratio, adult longevity, and diapause of *A. orientalis* wasps. The interaction between generation and photoperiod had a significant effect on female longevity and female fecundity, but there was no significant interaction between generation and photoperiod on male longevity. When reared under the long photoperiod condition (L16:D8), the third generation of *A. orientalis* had greater fecundity, a larger number of progenies, and a higher proportion of females than the population of wasps reared under the other photoperiods or the other generations of wasps. These results show that transgenerational photoperiods affect the biological traits of *A. orientalis*.

Studies to date have shown that various factors are important in regulating the fertility and development of *Anastatus* species. The key role of the natal host, photoperiod, temperature, and other environmental factors in the regulation of sex allocation, fertility, and adult longevity of *Anastatus* has been clearly demonstrated in both field observations and experimental studies (36–38). In addition, endogenous factors, such as their maternal age, parental experience, food plant of the host prey, and individual size, can also influence the resulting biological characteristics in certain *Anastatus* species (39). Our results suggest that photoperiod conditions and parental experience significantly impact longevity, female fecundity, sex ratio, and diapause rate of *A. orientalis* progeny. Host egg nutrient contents can affect sex ratio, adult longevity, and fecundity of a parasitoid’s progeny. Bai et al. (40) showed that females of *Trichogramma pretiosum* (Hymenoptera: Trichogrammatidae) from natural hosts were larger, more fecund, and lived longer than those from factitious hosts. Prior studies have found that *A. pernyi* eggs with higher nutrient contents are preferred and consumed, and this results in longevities reaching up to 64.3 days (25). Prior studies found that females can have a longevity of anywhere from 39 to 68 days in laboratory rearing settings (22, 23). In addition, findings show that larger parasitoids emerge when they develop in larger or more nutritious host eggs. Larger-sized parasitoid progenies show increased longevity, and larger-sized females are capable of higher oviposition rates. Together, these findings and this study show that *A. pernyi* is a suitable alternative host for rearing of the parasitoid.

Although temperature and photoperiod are known to induce diapause in most insect natural enemies, the results of this study indicate that the diapause in *A. orientalis* is induced by photoperiod alone. Seo et al. (22) tested the effect of temperature on biological characteristics of *A. orientalis*, but did not find any correlation between temperature and its diapause. Results by Broadley et al. (23) showed that a long photoperiod induces *A. orientalis* to enter diapause. Our experimental results show that diapause of *A. orientalis* is affected by its exposure to photoperiod, with wasps that experience a longer photoperiod showing a stronger prosperity for entering diapause. Therefore, we conclude that photoperiod is an important environmental factor for diapause induction in this parasitoid wasp. In addition, generation also had a significant effect on the diapause of *A. orientali*s; it increased in later generations. Only by surveying more successive generations can we verify this trend. These results provide additional information for viable long-term storage methods for *A. orientalis*, which in turn will improve rearing efficiency.

The interactive effect of temperature and photoperiod on diapause regulation has been documented in many insect species. For example, development of *Chrysocharis pubicornis* larvae (Hymenoptera: Eulophidae) showed that a short-day-type response affected by temperature and the percentage of individuals entering diapause increased with the temperature and photoperiod (41). In addition, results by Li et al. (42) suggest that the diapause response of *Microplitis mediator* (Hymenoptera: Braconidae) is determined by photoperiod and mediated by temperature. When *M. mediator* were exposed to 16°C and 18°C combined with a photoperiod of L10:D14, the percentages of parasitoids that entered diapause was 97.9% and 87.8%, respectively, and there was no incidence of diapause at temperatures of 22°C, 24°C, and 26°C or photoperiods of L2:D22, L14:D10, L16:D8, L18:D6, L20:D4, or L22:D2. Therefore, we are now performing experiments to study the effects of photoperiod and temperature, as well as the interaction of the two, on induction of diapause of *A. orientalis*.

Similar to Broadley et al. (23), but in contrast to work done by other scientists (22, 25), we were unable to produce non-diapause *A. orientalis* adults under the long photoperiod. This may be due to the strain of *A. orientalis* used between these different studies. Wu et al. (43) used molecular tools to examine the genetic composition of *A. orientalis*, and the results suggest a genetic component in determining the diapause behaviors of *A. orientalis*. In addition, Broadley et al. (44) determined that the *A. orientalis* used previously in their study (23), which also went into diapause when exposed to long photoperiod conditions, was a homogenous colony composed of haplotype C. Because the wasps in this study responded similarly, it is highly likely that specimens from our study are composed primarily of haplotype C. However, the different results across studies also may be due to the differences in colony rearing protocol or potentially in how transgenerational effects were expressed. Hence, several generations preceding the experiment should be kept under strictly controlled constant conditions, as this helps to evaluate such discrepancies. Similar precautions to exclude multigenerational maternal effects should be considered in experimental studies with this species and other insect species.

Storage time plays a key role in the production of *Anastatus* species, in that a longer storage time can provide a flexible supply of parasitoids for their timely and urgent field release in biological control programmes. The most commonly used preservation method for an egg parasitoid is cold storage (45–48). However, this method not only fails to guarantee its shelf life but also affects the quality of natural enemy products (23, 49, 50). According to our study’s results, diapause may be a better way to preserve *A. orientalis*, and rearing at long-day conditions can induce diapause. In addition, individuals that experienced diapause exhibited a higher sex ratio and greater fecundity, two traits that can augment its performance as a biological control agent. In other insect species, diapause has a positive effect on post-diapause adults’ fertility and development (51, 52). However, although diapause allows insects to cope with adverse environmental conditions, it also poses substantial fitness costs. Ellers et al. (53) showed that an increase in diapause length not only led to higher mortality among diapausing pupae of *Asobara tabida* (Hymenoptera: Braconidae), but also caused a significant decrease in egg load, fat reserves, and dry weight of the emerging adult females. Carvalho (54) results suggested that individuals experiencing diapause of *Utetes anastrephae* (Hymenoptera: Braconidae) have lower fecundity. Therefore, the correlation between diapause and reproductive or developmental traits varies with insect species.

Our study clearly shows that transgenerational experience can have far-reaching effects in subsequent generations. Unfortunately, it is not yet known how such effects are mediated or how grandmaternal effects in isolate of material effects would affect the outcome, but future studies are planned. Temperature conditions experienced transgenerationally may alter fertility and development of *A. orientalis*, but further study is needed (55). The empirical data from this study on the effects of transgenerational photoperiod experience on insect fertility and development have potential applications for better understanding *A. orientalis* population dynamics in the field across a geographic and climatic range, and improving insect rearing and storage methods.

## Data availability statement

The original contributions presented in the study are included in the article/**Supplementary Material**. Further inquiries can be directed to the corresponding author.

## Author contributions

All authors conceived, facilitated, and designed the research. K-XB conducted the experiments, analyzed the data, and conducted statistical analyses. X-YW and BX wrote the manuscript. X-YW, L-MC, HB, and JG secured funding. All authors contributed to the article and approved the submitted version.
